# Review of Deep Learning-Based Atrial Fibrillation Detection Studies

**DOI:** 10.3390/ijerph182111302

**Published:** 2021-10-28

**Authors:** Fatma Murat, Ferhat Sadak, Ozal Yildirim, Muhammed Talo, Ender Murat, Murat Karabatak, Yakup Demir, Ru-San Tan, U. Rajendra Acharya

**Affiliations:** 1 Department of Electrical and Electronics Engineering, Firat University, Elazig 23000, Turkey; ydemir@firat.edu.tr; 2 Department of Mechanical Engineering, Bartin University, Bartin 74100, Turkey; fsadak@bartin.edu.tr; 3 Department of Software Engineering, Firat University, Elazig 23000, Turkey; ozalyildirim@firat.edu.tr (O.Y.); mtalo@firat.edu.tr (M.T.); mkarabatak@firat.edu.tr (M.K.); 4 Department of Cardiology, Gülhane Training and Research Hospital, Ankara 06000, Turkey; ender.murat@sbu.edu.tr; 5 Department of Cardiology, National Heart Centre Singapore, Singapore 169609, Singapore; tanrsnhc@gmail.com; 6 Department of Cardiology, Duke-NUS Graduate Medical School, Singapore 169857, Singapore; 7 Department of Electronics and Computer Engineering, Ngee Ann Polytechnic, Singapore 138607, Singapore; aru@np.edu.sg; 8 Department of Bioinformatics and Medical Engineering, Asia University, Taichung 41354, Taiwan; 9 Department of Biomedical Engineering, School of Science and Technology, Singapore University of Social Sciences, Singapore 599494, Singapore

**Keywords:** atrial fibrillation, ECG, deep learning, deep neural networks, arrhythmia detection

## Abstract

Atrial fibrillation (AF) is a common arrhythmia that can lead to stroke, heart failure, and premature death. Manual screening of AF on electrocardiography (ECG) is time-consuming and prone to errors. To overcome these limitations, computer-aided diagnosis systems are developed using artificial intelligence techniques for automated detection of AF. Various machine learning and deep learning (DL) techniques have been developed for the automated detection of AF. In this review, we focused on the automated AF detection models developed using DL techniques. Twenty-four relevant articles published in international journals were reviewed. DL models based on deep neural network, convolutional neural network (CNN), recurrent neural network, long short-term memory, and hybrid structures were discussed. Our analysis showed that the majority of the studies used CNN models, which yielded the highest detection performance using ECG and heart rate variability signals. Details of the ECG databases used in the studies, performance metrics of the various models deployed, associated advantages and limitations, as well as proposed future work were summarized and discussed. This review paper serves as a useful resource for the researchers interested in developing innovative computer-assisted ECG-based DL approaches for AF detection.

## 1. Introduction

Atrial fibrillation (AF) is the most common heart rhythm disorder. It is seen mostly in the elderly but even young people who do not have underlying heart disease may suffer from it. Although AF itself is rarely lethal, it increases the risk of AF-related complications like heart failure and thromboembolism, which lead to increased morbidity and mortality [1]. AF is associated with a five and three times increase in risks of incident stroke [2] and heart failure [3], respectively. AF currently affects 33.5 million people globally, a number that is expected to increase rapidly due to population aging [4]. According to Gillis [5], the number of AF patients in the United States is expected to increase 2.5 times in the next 50 years. To avert AF complications and premature death, it is important to detect AF at an early stage to initiate appropriate preventive therapy, e.g., anticoagulation for cardio embolic stroke prevention.

AF can be classified as paroxysmal, persistent, or permanent AF [6,7]. Paroxysmal AF is an episode that lasts seven days or less. Persistent AF lasts more than seven days and necessitates additional therapy to terminate the episode, e.g., pharmacological or electrical cardioversion [8,9]. In permanent AF, therapy to cardiovert the rhythm is not attempted [10]. Regardless of their duration, all three classes of AF are associated with increased thromboembolic risks. Hence, accurate detection of AF episodes, however transitory, and initiation of anticoagulation are key to minimizing downstream adverse events.

The diagnosis of AF requires electrocardiographic (ECG) documentation of the arrhythmia on at least one lead [11]. Paroxysmal AF is easily missed on opportunistic office ECG recordings. Portable ambulatory extended duration ECG monitoring, e.g., 24-Holter recording, increases the odds of AF detection but generates voluminous ECG data that are onerous and time-consuming to analyze. An algorithm that can automatically pinpoint the onset and quantify the duration of AF episodes will have diagnostic and prognostic utility. The application of advanced signal processing and machine learning techniques to AF detection can help to reduce subjectivity and human error as well as improve the accuracy and timeliness of diagnosis [12].

Systems developed for automatic recognition of AF primarily exploit two key features of AF on ECG signals: absent P wave and/or irregular RR intervals. As such, accurate detection of P or R wave peaks is critical. The low amplitude P wave is especially susceptible to interference from ECG baseline drift and artifacts [13], which may lead to degraded performance of P wave-based algorithms [14,15] with noisy data signals. Another important method for determining the characteristics of ECG signals is heart rate variability (HRV). The increase in the spectral energy of HRV dynamics is a critical finding in the diagnosis of arrhythmia. One of the reasons they are preferred is their durability in noisy environments. Furthermore, HRV-based features only encode dynamic cardiac activity features. As a result, it has become one of the preferred methods in recent years, particularly for AF detection and PAF prediction.

Traditional machine learning algorithms are commonly used for ECG signal analysis [16,17]. The features representing cardiac arrhythmia are created in traditional machine learning techniques, usually as a result of interaction with experts and a literature review; they serve as inputs to shallow classifiers such as neural networks [16], SVM [17], KNN, and so on. These classifiers detect AF from ECG signals by using distinctive features. In the work of Henzel et al. [18], four statistical features of the RR interval were fed to a generalized linear classifier to diagnose AF. Overfitting on training data is a common weakness of these algorithms that rely on handcrafted feature extraction, which perform poorly when run on unseen data. In contrast, deep learning (DL) techniques incorporate automatic feature extraction and selection processes within the model. The computer can learn and extract related features in any problem automatically, which enhances the generalizability of DL models and renders them superior to traditional machine learning algorithms [19,20,21,22]. Convolutional neural networks (CNNs) are widely used in DL models for the analysis and classification of ECG signals [23,24,25,26,27,28,29]. CNNs can automatically learn representative complex features directly from the data itself, thereby eliminating the need for handcrafted features. Acharya et al. built an 11-layer CNN structure with a four neuron-output layer for ECG signal classification [30] and also another 11-layer CNN model that could distinguish shockable versus non-shockable ventricular arrhythmia [31]. In the work of Rahhal et al. [32], an unsupervised DL approach for ECG classification showed promising results when validated against well-known public databases such as MIT-BIH and INCART arrhythmia databases. In the work of Zubair et al. [33], CNNs for classifying ECG beats into five different classes were validated using 44 ECG recordings from the MIT-BIH database. A DL method called greedy deep dictionary learning [34] outperformed traditional and other DL methods. In [35], a new deep belief networks method that encompassed ECG signal pre-processing, segmentation and resampling, feature learning, and validation was able to learn the features of ECG arrhythmia and successfully classify them into five classes. By eliminating the need for manual feature extraction, the examples reviewed in this article underscore the generalizability and potential of DL models for detecting arrhythmia like AF on raw ECG signals [36,37,38,39].

We aimed to survey articles that have been published in international peer-reviewed journals on AF detection using DL, focusing on those that address the AF problem directly rather than generic studies of arrhythmia classification that included AF as one of the classes. The studies utilizing only DL in the detection of AF were examined in this study. Performance comparisons were made by considering the data sets, input formats, deep models, and classification approaches used in these studies. As a result, it is expected that researchers have knowledge in deep learning-based studies to be conducted for AF detection. Limitations of the studies and suggestions for future works for AF detection using DL were also discussed.

## 2. Materials and Methods

**Figure 1:**
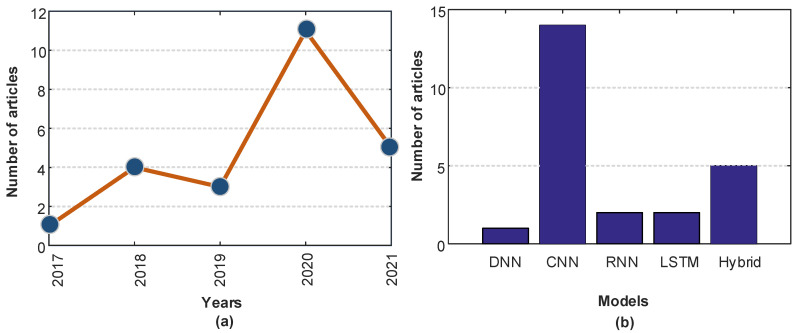
Distribution of publications on atrial fibrillation detection using deep learning by year of publication (**a**) and type of model deployed (**b**). CNN, convolutional neural network; DNN, deep neural network; LSTM, long short-term memory; RNN, recurrent neural network.

During the development of the search strategy, frequently used keywords in recent studies for the detection of AF were filtered. A review of the literature was conducted by searching the most used keywords (Atrial fibrillation detection, Arrhythmia detection, 12-Lead ECG, Atrial activity signal, etc.) and deep learning models (CNN, DNN, LSTM, etc.) and the search strategy was restricted to the last 5 years. The keywords “Atrial fibrillation and deep learning”, “Atrial fibrillation AND deep neural networks”, “Atrial fibrillation AND convolutional neural networks OR CNN”, “Atrial fibrillation AND LSTM”, and “Atrial fibrillation AND Neural networks” were used to search Google Scholar, Mendeley, and ScienceDirect databases for relevant articles. In total, 24 articles were selected that had been published in the following journals: Computers in Biology and Medicine [40,41,42,43], Journal of Electrocardiology [44], IEEE Journal of Biomedical and Health Informatics [45,46], Lancet [47], International Journal of Cardiology [48,49], Neural Computing and Applications [50], Biomedical Signal Processing and Control [51,52,53], Computer Methods and Programs in Biomedicine [54], Medical & Biological Engineering & Computing [55], Information Sciences [30,56], Expert Systems with Applications [19], Journal of Signal Processing Systems [57], AMIA Joint Summits on Translational Science proceedings, AMIA Joint Summits on Translational Science [58], Knowledge-Based Systems [59,60], and Future Generation Computer Systems [61]. Figure 1 shows the distribution of articles by year of publication and the DL model deployed.

**Figure 2:**
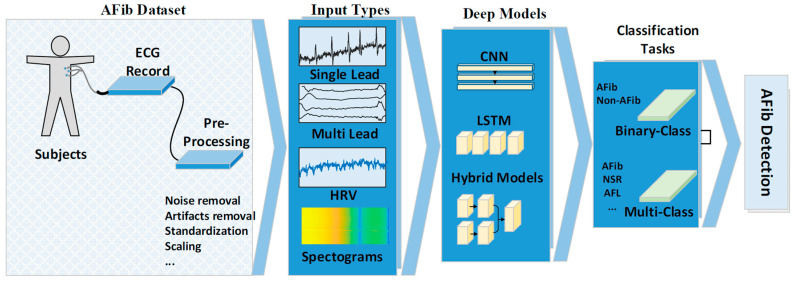
Block diagram representation of the general approach for deep learning-based atrial fibrillation detection.

From a detailed analysis of the various methods used in the articles, we constructed a general approach that is illustrated in Figure 2. The datasets used, the way the ECG signals were fed to the models, DL models used, and their classification approaches are discussed in the following section.

### 2.1. AF Datasets

Table 1 lists the ECG databases studied in the published papers. Among them, MIT-BIH DB [62], MIT-BIH AFDB [63], PhysioNet/CinC 2017 [64], MIT-BIH SRDB [62], MIT-BIH VFDB [62], and CU VTDB [65] were most commonly used.

#### Pre-Processing

An ECG signal often contains noise and artifacts that arise from the device used to collect the signal or the environment in which the signal is being collected. Various pre-processing techniques can be applied to denoise ECG signals, including Fourier cosine series operation to remove baseline wander and high frequency components [48], elliptical band-pass filter [40,43], wavelet transform [51,59,60,61,66], finite impulse response filter [45], band-pass Butterworth filter [42,52,55,66], and notch filter [67]. Additionally, to standardize the ECG signals for analysis, Z-score normalization [43,51,60] and high-pass filter [46] are commonly used for amplitude scaling and minimize offset effects, respectively.

### 2.2. Model Input Types

**Figure 3:**
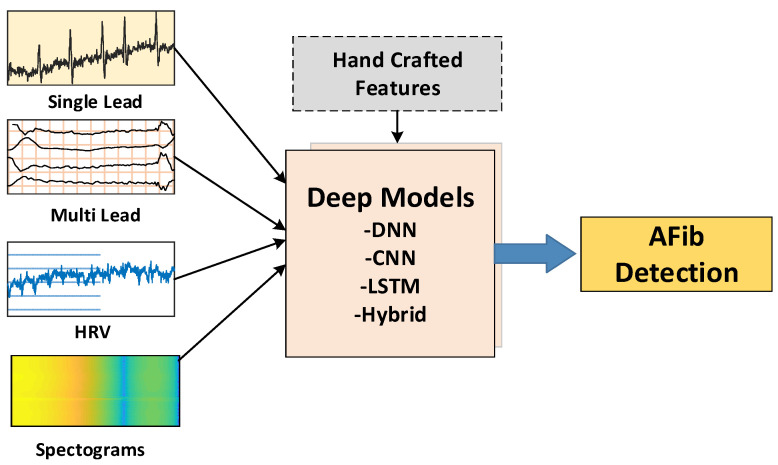
Block representation of ECG signal formats that can be input to deep learning models for atrial fibrillation detection.

The ECG signal can be configured in various formats—single-lead ECG, multi-lead ECG, heart rate variability (HRV), spectrogram, or fused features—for input into DL models for AF detection (Figure 3).

***Single-lead ECG:*** Single-lead input is commonly used in the published studies [19,30,42,45,48,51,52,53,55,56,60,61] as it is computationally lightweight, which facilitates model training. Lead II depicts the P, QRS, and T waves to good advantage [68] and which are used in many single-lead ECG input studies. The PhysioNet/CinC 2017 dataset comprising modified Lead I ECG signals acquired using a medical-grade portable personal ECG monitoring device has also been investigated in other AF studies.

***Multi-lead ECG:*** Studies using this data input generally have access to standard 12-lead ECG signal recordings [47]. As the data dimensionality is inordinately high for 12-lead ECG signals, some researchers used only a subset of 12-lead recordings to minimize the computational cost. Attia et al. [47] excluded four lead signals that contained little added information and used only eight leads (I, II, V1-6) as inputs for their DL model. Similarly, Baalman et al. [48] trained their model using only Lead II of the 12-lead ECG dataset.

***ECG segment size:*** Different segment sizes of single- and multi-lead ECG recordings have been used in the studies. Single-beat [48,54], five-second [42,59], and ten-second ECG signal segments [43,47,49] are common inputs. Fan et al. [45] compared the performance of 5-second, 10-second, 20-second, and 30-second ECG segment inputs in their DL model, and observed the best results with the 20-second segment input.

***Heart rate variability:*** HRV, which measures RR interval variations over a specified finite time duration, reflects the state of the autonomic nervous system [69,70] and has been extensively studied as model input for AF detection (Table 2). Faust et al. [41] segmented 100 beats with a floating window and input the resulting blocks which encompassed HRV information into a DL system to detect AF. This approach was validated using data from a different source in the work of Faust et al. [71].

Ebrahimzadeh et al. [77] extracted a total of 28 features from HRV signals, including nine linear features, 5 in the time domain and 4 in the frequency domain, 11 time-frequency features that include both time and frequency information, and 8 nonlinear features in each section. Similarly, Boon et al. [78] used time domain, frequency domain, and nonlinear analysis to extract 55 features from HRV.

***Spectrogram:*** One-dimensional signals like ECG RR intervals but not the ECG morphology can be converted to spectrograms [80,81,82] that have been used as inputs to DL models for AF detection [40,44,57]. Xia et al. [40] used short-time Fourier transform and stationary wavelet transform to convert five-second ECG segments into two-dimensional data. Rubin et al. [44] used fast Fourier transform on 85% overlapping 250-millisecond moving windows to convert one-dimensional ECG time series to time-frequency representations.

***Fused Features:*** Architectures with two or more different input types have been used for AF detection to improve model performance. In the work of Fan et al. [50], both RR interval information and ECG waveform morphological features were fed to two-layer fully connected networks to distinguish AF, sinus rhythm, and other arrhythmias. Lai et al. [46] used raw ECG data, fibrillatory wave spectra, and RR interval as inputs into their AF detection model. Tran et al. [58] proposed a DL network MultiFusionNet that combined two deep neural networks trained on different information sources using multiplicative fusion. In the work of Chen et al. [54], the proposed AF detection model combined CNN with its efficient automated learning and key feature extraction using both a recursive complex network [83] and coherence spectrum [84], which required additional manual features.

### 2.3. Deep Models

Table 3 lists the DL models developed for automatic AF detection. The most popular was CNN followed by a hybrid model that combined CNN and LSTM.

#### 2.3.1. Deep Neural Networks

Deep neural networks (DNNs), the most basic form of DL, have similar structures to the traditional multilayer perceptron (MLP) that is obtained by cascading models with multiple hidden layers. Learning is achieved by abstracting data inputs into the DNN’s many layers. Cai et al. [43] proposed a one-dimensional deep densely connected neural network comprising four blocks of multiple densely connected convolutional layers each, with a novel filter combination and unique use of squeeze and excitation module to enhance the network’s representation power. The model was able to accurately diagnose AF in binary and triple classification experiments using ten-second raw 12-lead ECG signals without the need to extract and select features.

#### 2.3.2. Convolutional Neural Networks

To learn, CNN models automatically extract hierarchical features from simple to complex using convolution by applying high-dimensional filters on the input data. They have been used successfully in problems involving two-dimensional images [85,86,87,88] as well as one-dimensional time-series data like ECG. CNN models used for AF detection can perform feature extraction and classification without the need for manual feature extraction. Xia et al. [40] were the first to use CNNs for AF detection. Unlike traditional AF-detection algorithms, their proposed method neither required manual feature extraction nor detection of ECG P and/or R waves. In the work of Fan et al. [45], a multi-scaled fusion of deep convolutional neural network (MS-CNN) employed two CNN streams each with 13 convolution layers and different filter sizes that could capture ECG features at different scales. After the max-pooling layer, the two streams were combined and the MS-CNN model completed with three fully connected layers. Fujita et al. [56] proposed a new system approach for AF and atrial flutter (AFL), an arrhythmia closely related to AF, detection using an eight-layer deep CNN. Using standard ten-second 12-lead ECGs, Attia et al. [47] built an artificial intelligence-enabled ECG machine that used a CNN model with a single convolution layer to detect AF. Fan et al. [50] proposed a CNN-based AF screening framework (FRM-CNN) to automatically screen for AF segments from mobile ECG signals using both ECG rhythm and morphological feature inputs. A 34-layer residual network was used to capture morphological features from ECG signals before both the morphological as well as rhythm features were input to a two-layer fully connected network with SoftMax layer for classification. Lai et al. [46] built four CNN models for classifying ECG data into AF and non-AF labels. Using different inputs, each model consisted of two convolution layers, two pooling layers, one batch-normalization, one fully connected, one input, and one output layer. Zhao et al. [57] proposed an 18-layer dense layered CNN model for AF detection. Wang et al. [60] combined a CNN and an improved Elman neural network (IENN), and created two linked models to validate the model’s classification performance. Among these last three models that differed in their decision mechanism for signal identification—MLP, Elman neural network (ENN), and IENN, respectively—but otherwise possessed similar structures, the IENN + CNN model [60] yielded the best performance for AF detection. Nurmaini et al. [61] proposed a one-dimensional CNN with two types of layers: (1) feature learning layers with one-dimensional convolutions and subsampling (pooling); and (2) fully connected layers as classifiers that are similar to the layers of a typical MLP. Different combinations of convolution and pooling layers were tested for classification performance. A 13-layered one-dimensional CNN model with five pooling layers—the more the pooling layers, the greater the reduction in model complexity—was found to have the best performance. Chen et al. [54] developed an accurate AF detection model that used two CNN algorithms to perform multi-feature extraction of atrial activity on ECG signals, which were combined with a decision-level fusion method. Despite working on a small training dataset without validation data, Nguyen et al. [53] were able to report better results than other common methods with their model, which combined CNN architecture for extracting deep features from ECG signal segments and a support vector machine (SVM) that classified each segment automatically without overfitting.

#### 2.3.3. Recurrent Neural Networks

Recurrent neural networks (RNNs) are a type of artificial neural network developed to solve temporal problems, particularly those with sequential inputs [89,90] such as ECG signals. Baalman et al. [48] fed single-cycle ECG morphological inputs to the attention mechanism of a RNN for AF detection. Of note, the use of single-cycle samples or short segments of ECG is especially suitable for real-time remote device/sensor monitoring applications. Mousavi et al. [42] input ECG signals to an attention mechanism of a bidirectional RNN (BiRNN). By increasing the number of attention mechanisms, four different models were created: the RNN model without the attention mechanism and three hierarchical attention network (HAN)-ECG models with one, two, and three attention mechanisms. Best accuracy and performance were obtained with more attention mechanisms, i.e., the HAN-ECG3 model, which contained wave attention, beat attention, and window attention layers sandwiched between BiRNN layers.

#### 2.3.4. Long Short-Term Memory

Long short-term memory (LSTM) models proposed by Hochreiter et al. [91] are widely used in DL to address deficiencies in the RNN architecture that include gradient exploding and vanishing problems, which limit the ability to learn lengthy-time period dependencies. The bidirectional LSTM designed by Faust et al. [41] effectively learned and extracted features from RR interval input data composed from 100-beat segments, and attained 98.51% and 99.77% accuracies for AF detection with ten-fold cross-validation and blindfold validation, respectively. The LSTM network was able to learn features in the presence or absence of AF that were then passed to the fully connected top model for classification, eliminating the need for information reduction by feature extraction. Cao et al. [51] used a two-layer LSTM network to train a public ECG database and reported that their proposed data augmentation method achieved a better F1 score for AF classification than without data augmentation.

#### 2.3.5. Hybrid Deep Models

There are theoretical synergies between CNN models’ representation learning and LSTM models’ sequence learning that can be combined to yield powerful DL models where features obtained from CNN layers are fed to LSTM layers in sequence. Andersen et al. [19] proposed an end-to-end model combining CNN and LSTM networks to classify ECG data as AF or sinus rhythm (SR) by extracting high-level features from RR intervals. Tran et al. [58] developed a deep structure incorporating both raw data and extracted features that captured the temporal dependence of the input data by including residual blocks and LSTM layers with the raw input data. Raw data were subjected to an average pooling layer in the CNN-LSTM model to mitigate long training times due to the large volume of data inputs. Jin et al. [59] proposed a twin-attentional convolutional LSTM neural network (TAC-LSTM) AF detection model that used CNN to compress ECG signals to obtain short-term characteristics and LSTM to obtain long-term dependency characteristics of ECG signals. Petmezas et al. [52] developed a deep CNN model to generate deep features from ECG signals followed by an LSTM layer for temporal dynamics memorization. They dealt with training data imbalance by employing focus loss, an improved version of cross-entropy loss, and reported success for detecting AF from four different rhythms. In Zhang et al. [55], ten-second ECG segments were input to the LSTM layer and the output fed to the CNN network to generate deep features that were finally classified by the SoftMax layer into AF versus non-AF labels. The training incorporated the Adam optimization method with a cross-entropy loss function. The proposed LSTM-CNN model showed good results when tested with three separate ECG databases.

### 2.4. Classification Task

The class to which ECG signal input belongs is determined at the final layers of DL models. Classification can either be binary—AF versus non-AF—or multi-class. In binary classification, the AF class may include AFL [42] and the non-AF class may include SR [40,41,43,45,47,48,55] and/or other arrhythmia [43,45,55,59]. In the multi-class approach, the ECG databases typically contain a variable number of classes besides AF, such as:

- AF, SR, AFL and ventricular fibrillation [30,56];
- AF, SR, and others [50];
- AF, SR, and AFL [60];
- AF, SR, others and noisy [44,51,53,57];
- AF, SR, and non-AF [61]; and
- AF, SR, AFL, and junctional rhythm [52].

## 3. Discussion and Comments

Table 4 summarizes the foregoing information on DL AF detection models. In general, more than 90% model accuracy for AF detection was attained. CNN models were the most popular [30,40,44,45,46,47,49,50,53,54,56,57,60,61]. In some of these studies, standard CNN layers were modified to networks of different sizes [30,46,47,49,56,61]. For example, Acharya et al. [30] achieved 92.50% and 94.90% accuracy rates for detecting AF on ECG segments of two different durations with an 11-layer CNN model. CNN AF detection models with 8 [56] and 13 layers [61] have also been proposed. Adding various feature extraction methods to the inputs of some CNN models was shown to enhance performance [40,44,50,54,57]. Inputting models with spectrograms containing time-frequency plots of ECG signals yielded good performance [40] without requiring manual feature extraction. The LSTM-based model proposed by Faust et al. [41] reported an excellent 99.77% accuracy with HRV signals, which underscores the potential for using HRV input in AF detection models.

CNN DL can perform automatic feature extraction effectively. Wang et al. [60] used the features obtained from the CNN model coupled with IENN classifiers and achieved high performance of 99.4%. Nguyen et al. [53] used the SVM classifier to classify the features obtained from the CNN layers and obtained a F1 score of 0.78 for AF detection. Representative features extracted from CNN layers can also be fed to LSTM models, which are effective at learning sequential features, with good results [52,58]. Conversely, when the LSTM architecture was used as a sequential feature extractor and the output was fed to the CNN model, lower performance was reported [55] compared with other studies.

Table 5 lists the studies based on the PhysioNet/CinC 2017 database, which may be relevant to potential mobile monitoring applications as the single-lead ECG signal data were acquired using a medical-grade portable device. Fan et al. [50] reported a very high F1 score of 0.88 for AF detection but the study did not include ECG signals in the noisy class. In studies where all four ECG classes in the database, sinus rhythm, AF, others, and noisy were included, AF detection F1 scores in a tight range of 0.78 to 0.84 were reported [44,51,53,57,58], with slightly better performance in the LSTM models [51,58].

This review chronicled research and development efforts to improve AF detection methodology through continual experimentation with network layer configuration and parameters. The observations contribute to the future design of DL models that are computationally efficient and yet can yield optimal results. Due to the blackbox nature of many DL models, there is a dearth of information on why a particular model should become successful or not. This constitutes an important impediment to clinical acceptance of new AF detection models [49], and a few studies have attempted to address this issue directly. Jo et al. [49] constructed saliency maps for the ECG that depicted the models’ explainability. Mousavi et al. [42] added to their model’s RNN backbone structure hierarchical attention mechanisms with interpretable transform effects on the detection results. Baalman et al. [48] developed a visualization tool for the attention vector that facilitated model interpretation. Lastly, disparities in the ECG signal input dimensions among the studies—ECG segments of different input sizes such as single beats [48,54], five- [42,59] and ten-second segments [43,47,48] have been used as input to the models—can limit the generalizability of the conclusions.

### Cardiologist Comments

It is important to distinguish AF from SR on the ECG. With manual interpretation, multi-lead ECG signals are more accurate than single-lead signals for AF diagnosis. For example, if the P wave in Lead II is positive and the P wave in precordial Lead V1 is negative or biphasic on a background of regular or equal RR intervals, SR is highly likely [92]. In theory, Leads II and V1 may represent the optimal two-lead ECG input combination for AF detection models that best balances accuracy with computational costs. Further, it would be appropriate to examine ECG segments lasting at least 30 s as the clinical significance of short episodes remains uncertain. By convention, AF on standard 12-lead ECG (the reference standard) and/or AF of at least 30 seconds in duration on any ECG recording are obligatory for the clinical diagnosis. Not surprisingly, DL models validated on 12-lead ECG database are arguably more credible and accurate. Nevertheless, single-lead ECG recordings are becoming more ubiquitous on personal and mobile devices and can no longer be dismissed as an increasingly relevant source of ECG signal data.

The chief motivation for developing AF screening systems is to detect AF accurately and reliably so that:

- Stroke and stroke-related complications can be prevented with early diagnosis of AF and initiation of oral anticoagulant therapy.
- AF-induced electrical and/or mechanical remodeling of the heart can be averted with rhythm and/or heart rate control.
- AF-associated heart failure can be prevented and/or ameliorated with specific heart failure drugs.
- AF-associated hospitalizations and healthcare expenditure can be reduced through optimal preventive management.

Possible limitations of AF screening include:

- Few public ECG databases are available for DL model training, which require a high volume of input data to develop accurate and robust models.
- Paroxysmal AF, which exacts similar stroke risk as persistent and permanent AF, may escape detection on 12-lead ECG and/or short-duration ECG monitoring.
- Related arrhythmia like AFL that are morphologically distinct from AF and yet also carries similar stroke risk as AF has only been included in selected studies.

## 4. Future Work

AF has effective preventive and therapeutic strategies and meets the criteria for cost-effective disease screening. Randomized controlled trials are incipient currently but interest is growing apace. When developing a DL model, consideration of the feasibility for implementation in cloud-based applications for real-world, real-time monitoring is imperative. Wearable technology provides low-cost and practical data input options for arrhythmia screening, and DL models are an efficient framework for signal analysis and interpretation.

**Figure 4:**
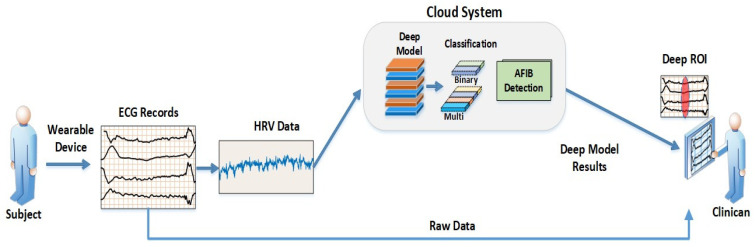
A block representation of cloud-based atrial fibrillation detection system using ECG records.

Figure 4 illustrates a proposed cloud-based AF detection system that can be employed on mobile phones. The HRV signal, which we showed to yield the best performance [41] among the studies reviewed, is extracted from ECG recording and sent to the cloud for processing. The processed data is interpreted by the cloud-based DL model and results are relayed back to the clinician with minimal human effort. After verification by the clinician, the vetted results are sent to the patient’s mobile phone. The feasibility of such a system is dependent on managing the computational costs of the DL model as well as data dimensionality. Of note, HRV signals occupy smaller bandwidths than ECG signals and can be acquired on mobile devices for real-time applications. Finally, the cloud-based system offers the optionality of processes to be conducted online or offline, which should garner clinical acceptance.

## 5. Conclusions

In this study, we discussed 24 papers on DL methods developed for automatic detection of AF on ECG-based signals. Most of the studies used CNN models, which yielded good results with ECG as well as HRV signals. This study can serve as a guide for researchers interested in designing optimal DL models for AF detection with the least computational costs. Aside from the limitations of deep learning methods (number of data, computational costs, etc.), another significant limitation of the study is the absence of a systematic search method. On some general journal search engines, the keywords determined were used to conduct a search. In future studies, this search strategy and journal search engines can be expanded to conduct a more systematic review.

## Figures and Tables

**Table 1:** EGG databases used in studies of atrial fibrillation detection based on deep learning.

Database	Records	Papers
MIT-BIH DB	0.5 h duration, 48 records from 47 subjects, 360 Hz sampling rate	[19,30,46,50,54,56,60]
MIT-BIH AFDB	10 h duration, 25 records, 250 Hz sampling rate	[19,30,40,41,42,52,55,56,59,60,61]
PhysioNet/CinC 2017	8528 single-lead ECG, 300 Hz	[42,44,45,49,50,51,53,55,57,58]
MIT-BIH VFDB	0.5 h duration, 22 records	[56]
CU VTDB	8 min, 35 records, 250 Hz sampling rate	[30]
Others	Details in individual papers	[43,46,47,48,49,54,55,61]

**Table 2:** Summary of studies performed on atrial fibrillation detection with heart rate variability signals.

Author, Year	Purpose	Classifier	Input	Performance (%)		
				Spec.	Sen.	Acc.
Faust et al., 2018 [41]	AF detection	Bidirectional LSTM	23 subjects	99.61	99.87	99.77
Mei et al., 2018 [72]	AF detection	SVM + BT	8528 single-lead ECG	98.6	83.2	96.6
Mohebbi et al., 2012 [73]	PAF prediction	SVM	30-min ECG	93.10	96.30	-
Narin et al., 2018 [74]	PAF prediction	KNN	5-min ECG	88	92	90
Chesnokov, 2008 [75]	PAF prediction	SVM	30-min segments	93	76	-
Hirsch et al., 2021 [76]	AF detection	BoT, RF, LDA	30-beat window	96.1	95.9	97.4
Ebrahimzadeh et al., 2018 [77]	PAF prediction	MLP, KNN, SVM	5-min ECG	95.55	100	98.21
Boon et al., 2016 [78]	PAF prediction	SVM	30-min ECG	79.3	81.1	80.2
Marinnucci et al., 2020 [79]	AF identification	ANN	8244 ECG	75.0	88.7	-

Acc, accuracy; AF, atrial fibrillation; ANN, artificial neural network; AUC, area under curve; BT, bagging tree; BoT, boosted trees; KNN, k-nearest neighbor; LDA, linear discriminant analysis; LSTM, long short-term memory; MLP, multilayer perceptron; PAF, paroxysmal atrial fibrillation; RF, random forest; Spec, specificity; SVM, support vector machine.

**Table 3:** Deep learning models developed for automatic AF detection.

Deep Models	Related Publications	Advantage/Disadvantage
DNN	[43]	In terms of speed, it is more advantageous.
CNN	[30,40,44,45,46,47,49,50,53,54,56,57,60,61]	Strong in obtaining representative properties, but lacking in design difficulties and parameter tuning.
RNN	[42,48]	Although it is used because of its memory structure, it is poor at representing sequences.
LSTM	[41,51]	Although useful for sequence representations, it is slow and consumes a lot of resources.
Hybrid (CNN+LSTM)	[19,52,55,58,59]	The use of both representation and sequence features together is advantageous, but it takes more time and cost.

CNN, convolutional neural network; DNN, deep neural network; LSTM, long short-term memory; RNN, recurrent neural network.

**Table 4:** Deep learning models for atrial fibrillation detection.

Authors, Year	Number of Subjects	Leads	Classes	Database	Method	Performance (%)		
						**Spec.**	**Sen.**	**Acc.**
Acharya et al., 2017 [30]	21,709 2 s ECG segments 8683 5 s ECG segments	Lead II	SR, AF, AFL and VF	MIT-BIH DB, MIT-BIH AFDB, CU VTDB	11-layer CNN	93.13 81.44	98.09 99.13	92.50 94.90
Xia et al., 2018 [40]	162,536 5 s ECG segments	2 Lead	AF and non-AF	MIT-BIH AFDB	STFT (RGB) + CNN STFT (grayscale) + CNN SWT + CNN	98.24 97.17 97.87	98.34 98.60 98.79	98.29 97.74 98.63
Faust et al., 2018 [41]	CV: 20 subjects BV: 3 subjects	-	SR and AF	MIT-BIH AFDB	HRV + bidirectional LSTM	98.67 99.61	98.32 99.87	98.51 99.77
Fan et al., 2018 [45]	5154 SR recordings 7713 AF recordings	Single Lead	SR and AF, AF and O	PhysioNet/CinC 2017	MS-CNN	98.77 98.84	93.77 80.26	98.13 97.19
Andersen et al., 2019 [19]	23 long-term recordings 48 short-term recordings 18 long-term recordings	Single Lead	SR and AF	MIT-BIH AFDB, MIT-BIH DB, MIT-BIH SRDB	CNN + LSTM	96.95 86.04 95.01	98.98 98.96 -	97.80 87.40 -
Fujita et al., 2019 [56]	25,287 2 s ECG segments	Single Lead	SR, AF, AFL and VF	MIT-BIH DB, MIT-BIH AFDB, MIT-BIH VFDB	8-layer CNN	96.07	99.43	98.61
Attia et al., 2019 [47]	649,931 10 s ECG recordings	12 Lead	SR and AF (includes AFL)	Mayo Clinic ECG Laboratory	CNN	83.4	82.3	83.3
Baalman et al., 2020 [48]	1499 10 s ECG recordings	Lead II, 8 Lead	SR and AF	AFACT	R-centered SC-ECG + RNN R-to-R-wave SC-ECG + RNN	-	-	94.00 96.00
Cai et al., 2020 [43]	16,557 10 s ECG recordings	12 Lead	SR and AF AF and non-AF SR, AF and O	Chinese PLA General Hospital Wearable 12-Lead, The China Physiological Signal 2018	DDNN	99.19 97.04 95.85	99.44 98.63 98.38	99.35 98.21 97.74
Lai et al., 2020 [46]	510,472 10 s ECG recordings	Multi Lead	AF and non-AF	Hexin Patch Lead II, MIT-BIH DB	8-layer CNN	93.4	93.1	93.1
Jin et al., 2020 [59]	150,060 5 s ECG recordings	-	AF and non-AF	MIT-BIH AFDB	Multi-domain feature + TAC-LSTM	98.76	98.14	98.51
Wang et al., 2020 [60]	22,174 ECG segments 1265 ECG segments	Single Lead	SR, AF and AFL	MIT-BIH AFDB, MIT-BIH DB	CNN + MLP CNN + ENN CNN + IENN	99.3 99.6	97.1 99.3	98.3 99.4
Nurmaini et al., 2020 [61]	6114 samples (9 s)	Single Lead	SR and AF SR, AF and non-AF	PhysioNet AFDB, MIT-BIH AFDB, MIT-BIH Malignant Ventricular Entropy, An Indonesian Hospital	13-layer one-dimensional CNN	99.91 99.17	99.91 98.90	99.98 99.17
Mousavi et al., 2020 [42]	167,422 5 s ECG recordings 8528 ECG recordings	Single Lead	AF and non-AF SR and AF	MIT-BIH AFDB, PhysioNet/CinC 2017	BiRNN (HAN-ECG)	98.54	99.08	98.81
Chen et al., 2021 [54]	-	2 Lead 12 Lead	SR and AF	MIT-BIH DB, AHA DB, QT DB, CSE DB	Multiple feature extraction + CNN	-	-	98.92
Petmezas et al., 2021 [52]	970,009 beats	2 Lead	SR, AF, AFL and J	MIT-BIH AFDB	CNN + LSTM + FL	99.29	97.87	-
Jo et al., 2021 [49]	-	12 lead, 6 Lead, Single Lead	AF and non-AF	Sejong ECG DB, PTB-XL ECG DB, Charman et al. ECG DB, PhysioNet DB	CNN	99.5	99.9	99.6
Zhang et al., 2021 [55]	80,000 ECG segments 83,464 ECG segments 19,220 ECG segments	Lead I	AF and non-AF	Wearable Lead I-II, MIT-BIH AFDB, PhysioNet/CinC 2017	LSTM + CNN	95.19 94.49 96.66	97.73 96.46 92.09	95.44 95.28 96.23

Acc, accuracy; AF, atrial fibrillation; AFL, atrial flutter; BiRNN, bidirectional RNN; BV, blindfold validation; CNN, convolutional neural network; CV, cross-validation; DDNN, deep densely connected neural network; ENN, Elman neural network; FL, focal loss; FRM-CNN, CNN-based AF screening framework; HAN, hierarchical attention network; HRV, heart rate variability; IENN, improved Elman neural network; J, junctional rhythm; LSTM, long short-term memory; MLP, multilayer perceptron; MS-CNN, multi-scaled fusion of deep CNN; N, noisy; O, others; RNN, recurrent neural network; SC, single-cycle; Sen, Sensitivity; Spec, Specificity; SR, sinus rhythm; STFT, short-time Fourier transform; SWT, stationary wavelet transform; TAC-LSTM, twin-attentional convolutional LSTM; VF, ventricular fibrillation.

**Table 5:** Deep learning models for atrial fibrillation detection using the PhysioNet/CinC 2017 dataset.

Authors, Year	Classes	Method	F_1N_	F_1A_	F_1O_	F_1_
Rubin et al., 2018 [44]	SR, AF, O and N	SQA + DCNN	0.91	0.83	0.72	0.82
Fan et al., 2020 [50]	SR, AF and O	FRM-CNN	0.93	0.88	0.74	0.85
Zhao et al., 2020 [57]	SR, AF, O and N	Kalman filter + DCNN	0.89	0.79	0.72	0.80
Tran et al., 2020 [58]	SR, AF, O and N	CNN + LSTM	0.90	0.83	0.75	0.80
Cao et al., 2020 [51]	SR, AF, O and N	2-layer LSTM	0.91	0.84	0.70	0.82
Nguyen et al., 2021 [53]	SR, AF, O and N	Stacking CNN + SVM	0.93	0.78	0.79	0.83

AF, atrial fibrillation; CNN, convolutional neural network; DCNN, densely connect neural network; F1, F1 score; FRM-CNN, CNN-based AF screening framework; LSTM, long short-term memory; N, noisy; O, others; SQA, signal quality analysis; SR, sinus rhythm; SVM, support vector machine.
